# A spatio-temporal atlas of the developing fetal brain with spina bifida aperta

**DOI:** 10.12688/openreseurope.13914.1

**Published:** 2021-10-15

**Authors:** Lucas Fidon, Elizabeth Viola, Nada Mufti, Anna L. David, Andrew Melbourne, Philippe Demaerel, Sébastien Ourselin, Tom Vercauteren, Jan Deprest, Michael Aertsen

**Affiliations:** 1 School of Biomedical Engineering & Imaging Sciences, King’s College London, London, SE1 7EU, UK; 2 Elizabeth Garrett Anderson Institute for Women’s Health, University College London, London, WC1E 6DB, UK; 3 Department of Obstetrics and Gynaecology, University Hospitals Leuven, 3000 Leuven, Belgium; 4 Department of Radiology, University Hospitals Leuven, 3000 Leuven, Belgium

**Keywords:** Fetal brain atlas, spina bifida aperta, fetal brain development, fetal brain T2-weighted MRI, anatomical landmarks, spatio-temporal atlas, segmentation, super resolution and reconstruction

## Abstract

**Background:** Spina  bifida  aperta  (SBA)  is  a  birth  defect  associated  with  severe anatomical changes in the developing fetal brain.  Brain magnetic resonance imaging (MRI) atlases are popular tools for studying neuropathology in the brain anatomy,  but  previous  fetal  brain  MRI  atlases  have  focused  on  the  normal  fetal brain. We aimed to develop a spatio-temporal fetal brain MRI atlas for SBA.

**Methods:** We developed a semi-automatic computational method to compute the first spatio-temporal fetal brain MRI atlas for SBA. We used 90 MRIs of fetuses with SBA with gestational ages ranging from 21 to 35 weeks. Isotropic and motion-free  3D  reconstructed  MRIs  were  obtained  for  all  the  examinations.   We propose a protocol for the annotation of anatomical landmarks in brain 3D MRI of fetuses with SBA with the aim of making spatial alignment of abnormal fetal brain MRIs  more  robust.   In  addition,  we  propose  a  weighted  generalized  Procrustes method based on the anatomical landmarks for the initialization of the atlas.  The proposed weighted generalized Procrustes can handle temporal regularization and missing annotations.  After initialization, the atlas is refined iteratively using non-linear  image  registration  based  on  the  image  intensity  and  the  anatomical  land-marks.  A semi-automatic method is used to obtain a parcellation of our fetal brain atlas into eight tissue types:  white matter, ventricular system, cerebellum, extra-axial  cerebrospinal  fluid,  cortical  gray  matter,  deep  gray  matter,  brainstem,  and corpus callosum.

**Results:** An intra-rater variability analysis suggests that the seven anatomical land-marks are sufficiently reliable. We find that the proposed atlas outperforms a normal fetal brain atlas for the automatic segmentation of brain 3D MRI of fetuses with SBA.

**Conclusions:** We make publicly available a spatio-temporal fetal brain MRI atlas for SBA, available here: https://doi.org/10.7303/syn25887675. This atlas can support future research on automatic segmentation methods for brain 3D MRI of fetuses with SBA.

## Plain language summary

Approximately five per 10,000 babies born in Europe suffer from spina bifida aperta (SBA). SBA is a birth defect that occurs when the spinal column of the fetus fails to close during the first month of pregnancy. SBA can impact the development of the fetal brain, resulting in lifelong disabilities such as cognitive impairment, difficulties with mobility, and a reduced life expectancy. The effect of SBA on the development of the fetal brain is complex and is not yet fully understood. Developing our visualization of SBA is fundamental to improving diagnosis and management for babies born with this condition. Fetal brain atlases are maps of the development of the fetal brain during the pregnancy. Such atlases allow researchers to perform measurements of the fetal brain anatomy and to study the development of the fetal brain in a large population. However, current fetal brain atlases only correspond to expected fetal brain development. In this work, we developed the first atlas of the developing brain in fetuses with SBA between 21 weeks and 34 weeks of gestation. This condition-specific atlas will allow us to perform more accurate measurements in fetuses with SBA. The atlas is created from 90 Magnetic Resonance Imaging (MRI) scans taken of fetuses with SBA in the womb, a technique which allows the fetal brain to be visualised in 3D and in high resolution. The period 21–34 weeks of the development of the fetal brain in SBA is of particular interest because surgery performed while the baby is still in the womb is currently completed prior to 26 weeks of gestation. The proposed atlas could therefore support research on the effect of the surgery on the fetal brain anatomy.

## 1 Introduction

Spina bifida aperta (SBA) is the most prevalent fetal brain defect with approximately five per 10,000 live births in Europe ^1^. It occurs when the neural tube fails to close in the first four weeks after conception. Most cases of SBA are accompanied by severe anatomical brain abnormalities ^2^ with enlargement of the ventricles and a type II Chiari malformation being most prevalent. The Chiari malformation type II is characterized by a small posterior fossa and hindbrain herniation in which the medulla, cerebellum, and fourth ventricule are displaced caudally into the spinal canal ^3^. The corpus callosum of fetuses with SBA is also abnormal ^2, 4^ and has been found to be significantly smaller for fetuses with SBA than for normal fetuses ^4– 6^. SBA fetuses have also smaller hippocampus ^7^, abnormal cortical thickness and gyrification ^8, 9^, and smaller deep grey matter volume and total brain volume ^10, 11^. For all those reasons the anatomy of the brain of fetuses with SBA differs from the normal fetal brain anatomy. In addition, the mechanisms underlying those anatomical brain abnormalities remain incompletely understood ^12^.

Brain atlases are used to study common trends and variations in the brain anatomy of a population. They provide a model of a population of brain magnetic resonance images (MRIs) that represents the average brain anatomy of a population, allow the comparison of measurements in a cohort study, and can be used for the automatic segmentation of brain MRIs ^13– 16^. Atlases can also be used to measure variability in the brain anatomy of an individual as compared to the whole population ^13^. Age and disease specific atlases allow a more accurate model of specific populations of human brains to be obtained ^17^.

Previous work on fetal brain atlases has focused on age-specific atlases by proposing various spatio-temporal fetal brain MRI atlases ^13, 14, 16, 18– 21^. A spatio-temporal atlas does not consist in only one average volume, but instead consists in a collection of age-specific average volumes. This allows the development of the fetal brain anatomy to be modelled. However, existing studies have only used brain MRIs of fetuses with a normal brain development, except for one study that combined fetuses with a normal brain and fetuses with lissencephaly in the same atlas ^13^. In particular, no fetal brain atlas for the developing fetal brain with SBA has been proposed in the literature.

In this work, we propose the first spatio-temporal fetal brain MRI atlas for SBA. Our atlas covers all the weeks of gestation between 21 weeks and 34 weeks. This range of gestational ages is of particular interest for SBA because it starts before the time at which in-utero surgery for SBA is currently performed ^12^ and covers most of the time until birth. The atlas is computed using 90 fetal brain MRIs from 37 fetuses with SBA. We hypothesise that the high variability of the brain anatomy in SBA is one of the main challenges in adapting methods developed for normal fetal brain atlases for SBA. To tackle this issue, we propose a semi-automatic method for the computation of the proposed fetal brain MRI atlas for SBA. We propose a protocol for the annotation of 11 anatomical landmarks in fetal brain 3D MRI of fetuses. Those anatomical landmarks are used for two things in our pipeline. The anatomical landmarks are used firstly to initialize the computation of the atlas using a weighted generalized Procrustes method and secondly to regularize the non-linear image registration of fetal brain 3D MRIs to the atlas.

We performed an intra-rater variability evaluation for the proposed landmarks using a subset of 31 3D MRIs from our cohort. Based on this evaluation, 4 anatomical landmarks were excluded and 7 were selected to help for the computation of the spatio-temporal atlas. In addition, we evaluated the automatic fetal brain segmentations computed using the proposed atlas for SBA on 40 fetal brain 3D MRIs of the publicly available FeTA dataset ^22^. It contains 15 MRIs of normal fetuses and 25 MRIs of fetuses with SBA. We compared the automatic segmentations computed using our SBA atlas to the segmentations computed using a state-of-the-art normal fetal brain MRI atlas ^19^. We have found that the proposed SBA atlas outperforms the normal fetal brain atlas on cases with SBA. The proposed spatio-temporal fetal brain MRI atlas for SBA is made publicly available here.

## 2 Materials

In this section, we describe the fetal brain MRI data used to compute the atlas and for the evaluation of automatic segmentations obtained using the atlas.

### 2.1 Ethics statement

The MRI data were automatically pseudonymized using the GIFT-Cloud data sharing platform ^23^ prior to using them for research.

At University Hospitals Leuven, ethical approval to use the data for research was given by the Ethics Committee University Hospitals Leuven (ethical approval S63598). A retrospective study does not fall under the Belgian law of May 7, 2004 regarding experiments on the human person. However, given the use of potentially identifying MRIs in the study, the requirements set forth in the EU Regulation 2016/679 (General Data Protection Regulation, GDPR) must be met. The sponsor of this study is University Hospitals Leuven, and University Hospitals Leuven maintains "public interest" as the legal basis for data processing. Article 14 of the GDPR mentions the information obligation of the data controller (= sponsor of the study) to the data subject whose personal data are collected. An information obligation is therefore sufficient according to GDPR, and informed consent is not legally required for the use of the MRIs for illustrative purposes. All snapshots of fetal MRIs used in our figures are based on MRIs acquired at Leuven.

At University College London Hospital (UCLH) the study was approved by the Caldicott guardian at UCLH and patient consent was not required as these images were acquired for clinical purposes and the data used retrospectively.

### 2.2 Spina bifida aperta cohort used to compute the spatio-temporal atlas

A total of 90 fetal brain MRI examinations from 37 fetuses were used in this work.

**Figure 1.**
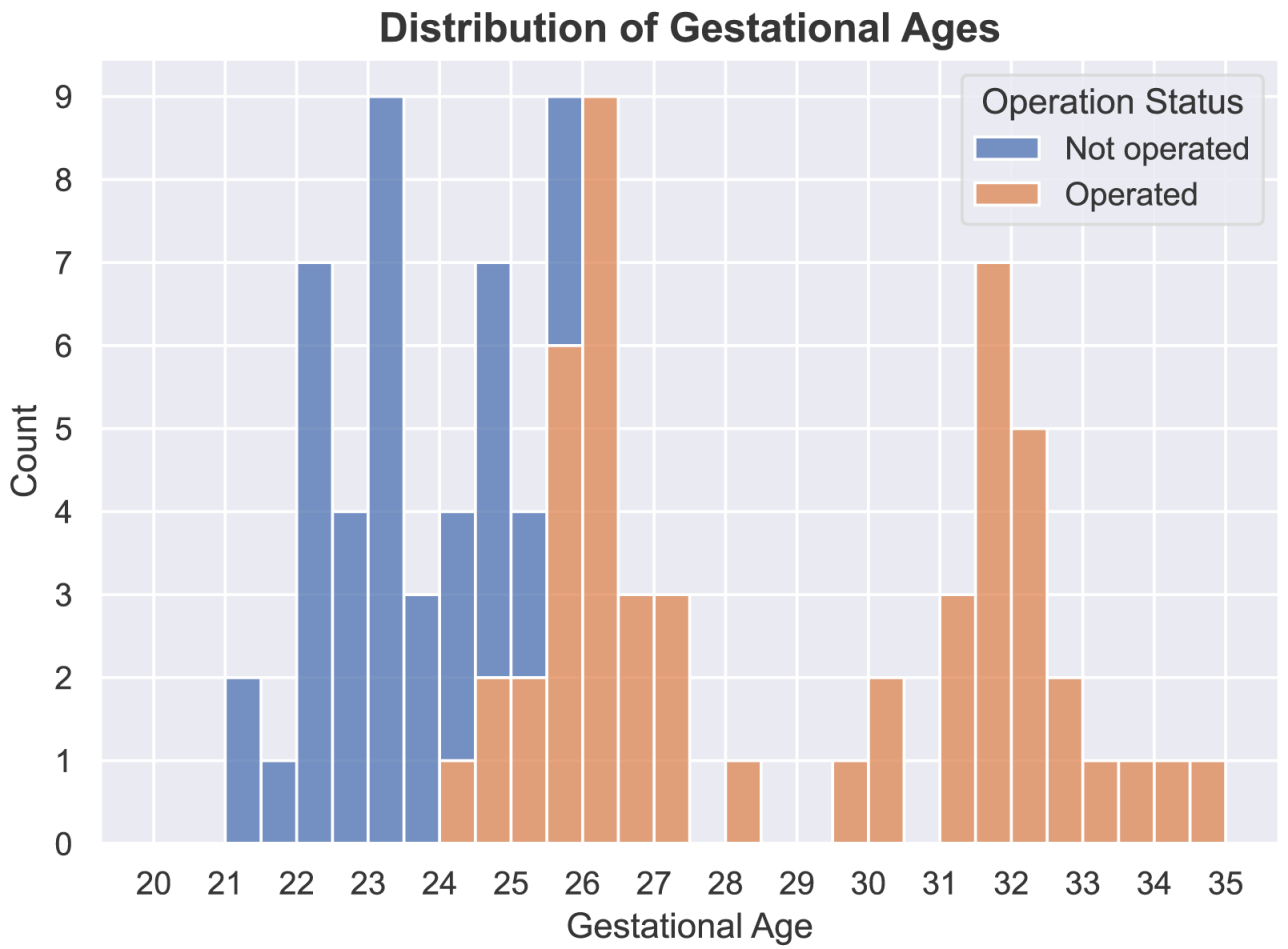
Distribution of gestational ages for operated (fetal surgery) and non-operated fetal brains. The dataset used to compute the atlas contains 39 magnetic resonance imaging (MRI) examinations of non-operated fetuses and 51 MRI examinations of operated fetuses.

All the MRI examinations were performed as part of clinical routine following abnormal findings during ultrasound examination. All the fetuses in this cohort were diagnosed with spina bifida aperta at fetal ultrasound examinations. MRI scans were acquired at two surgical centers, University Hospitals Leuven and UCLH (see *Underlying data*). For each study, at least three orthogonal T2-weighted HASTE series of the fetal brain were collected on a 1.5T scanner using an echo time of 133ms, a repetition time of 1000ms, with no slice overlap nor gap, pixel size 0.39mm to 1.48mm, and slice thickness 2.50mm to 4.40mm. A radiologist attended all the acquisitions for quality control. The dataset contains longitudinal MRI examinations with up to 5 examinations per fetus. In addition, 51 of the MRI examinations were performed after open fetal surgery performed before 26 weeks of gestation, to close the spina bifida aperta defect. The distribution of gestational ages for MRI examinations and whether they were done before or after surgery can be found in Figure 1.

### 2.3 Fetal brain 3D MRI used for the evaluation of automatic segmentation

For the evaluation of automatic fetal brain segmentation we have used the publicly available FeTA dataset ^22^ (first release).

The FeTA dataset contains 40 reconstructed 3D MRIs, including 15 MRIs of fetuses with a normal brain and 25 MRIs of fetuses with spina bifida aperta. For all the 3D MRIs, segmentations are available for seven tissue types: white matter, ventricular system, cerebellum, extra-axial cerebrospinal fluid, cortical grey matter, deep grey matter, and brainstem. The 40 3D MRIs and original segmentations (as provided with the FeTA dataset) were inspected by two paediatric radiologists within our institutions, MA and PD, with more than 8 years of experience in segmenting fetal brains. Corrections of the segmentations were performed ^24, 25^ to reduce the variability against the published segmentation guidelines that was released with the FeTA dataset ^22^. Two volumes of spina bifida aperta cases ( sub-feta007 and sub-feta009) were excluded because the poor quality of the 3D reconstruction did not allow to segment them reliably for the seven tissue types.

### 2.4 Spatio-temporal atlas for the normal developing fetal brain

For comparison to a spatio-temporal atlas of the normal developing fetal brain, we have used the publicly available spatio-temporal fetal brain atlas ^19^. This atlas contains 18 3D MRIs of average normal fetal brain for gestational ages ranging from 21 weeks to 38 weeks.

## 3 Atlas computation method

**Figure 2.**
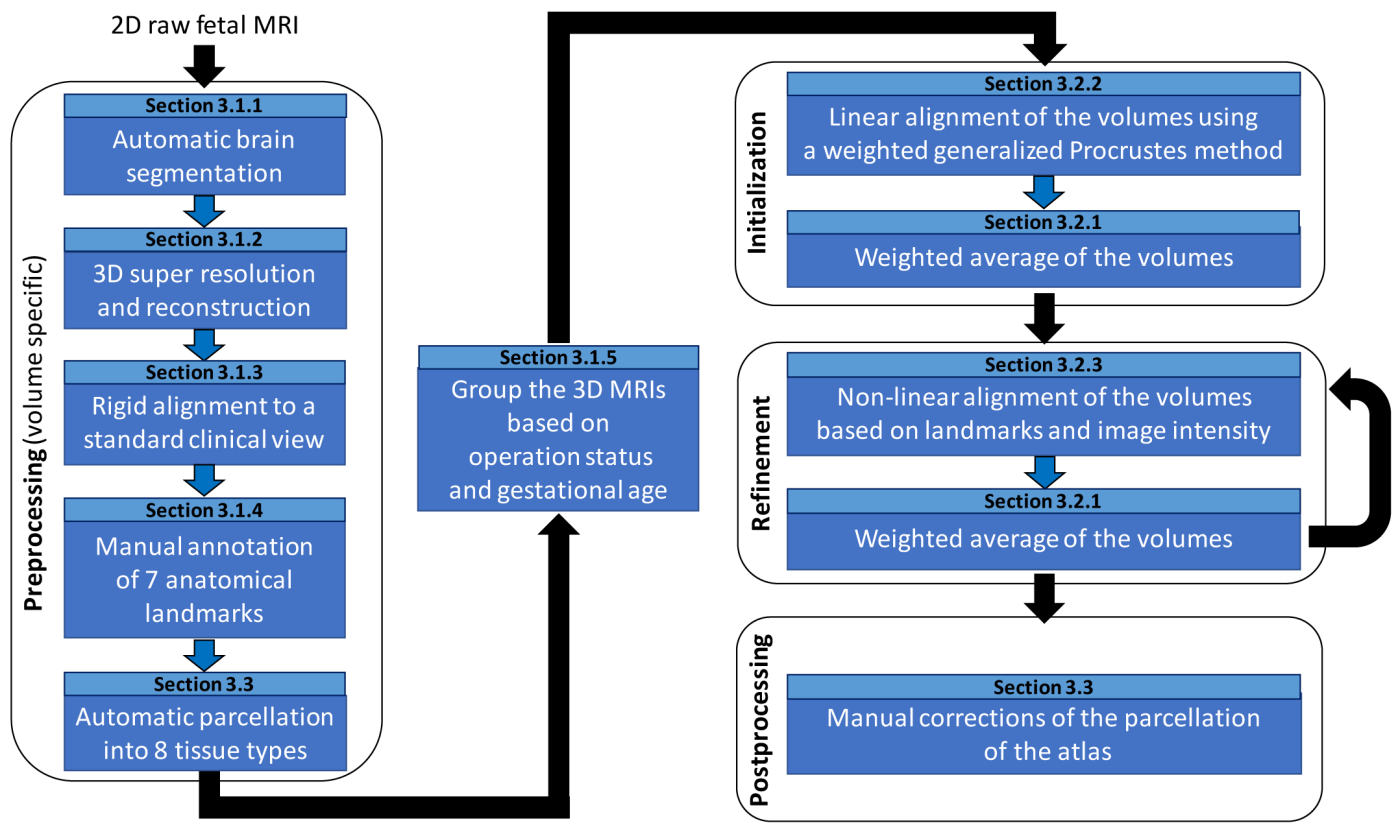
Overview of the spatio-temporal atlas construction pipeline. MRI: magnetic resonance imaging.

In this section, we describe our pipeline for computing the spina bifida aperta (SBA) fetal brain 4D atlas. An overview of the pipeline can be found in Figure 2.

### 3.1 Data preprocessing

In this subsection, we give details about the preprocessing steps as can be found in Figure 2.

***3.1.1 Automatic brain segmentation.*** One of the main challenges in fetal brain MRI is the motion of the fetus. To tackle this issue, MRI sequences used for fetal MRI are designed to produce multiple stacks of 2D slices rather than a 3D image. Original 2D slices typically have lower resolution, suffers from motion between neighboring slices, motion artefact, and suboptimal cross-section ^26^. Automatic segmentation of the fetal brain in the raw 2D MRI are obtained using a deep learning-based method ^27^. Those brain masks are an input required by the 3D super resolution and reconstruction algorithm described below. A public implementation of the deep learning pipeline MONAIfbs ^27^, used in this study to obtain the brain masks, can be found here (main git branch, commit *bcab*52 *a*).

***3.1.2 3D super resolution and reconstruction.*** We use a 3D super resolution and reconstruction algorithm to improve the resolution, and remove motion between neighboring slices and motion artefacts present in the original 2D slices ^26^. The output of the 3D super resolution and reconstruction algorithm ^26^ is a reconstructed 3D MRI of the fetal brain with an isotropic image resolution (of 0.8 mm in our case). We hypothesize that the reconstructed 3D MRI facilitates the manual delineation and annotation of the fetal brain structures as compared to the original 2D slices.

We used a state-of-the-art 3D super resolution and reconstruction algorithm ^26^ publicly available in the NiftyMIC pipeline version 0.8 with Python 3.8. The original 2D MRI slices were also corrected for bias field in the NiftyMIC pipeline version 0.8 using a N4 bias field correction step as implemented in SimpleITK version 1.2.4. The 3D super resolution and reconstruction algorithm ^26^ also combines the brain masks obtained in section Automatic brain segmentation. This results in a 3D brain mask for the 3D reconstructed MRI that is computed fully-automatically.

***3.1.3 Rigid alignment to a standard clinical view.*** The 3D reconstructed MRI were rigidly aligned to a time-point volume of the control fetal brain 4D atlas ^19^ as implemented in NiftyMIC ^26^ version 0.8. All the 3D reconstructed MRIs are therefore aligned to a standard clinical view in which the axes are aligned with the axial, sagittal, and coronal planes of the fetal brain. This facilitates the manual delineation and annotation of the fetal brain structures. The target time-point in the control 4D atlas is chosen based on the brain volume computed using the automatic 3D brain mask.

***3.1.4 Anatomical landmarks.*** Seven anatomical landmarks were manually annotated to regularize and improve the accuracy of the image registration steps used in the computation of the spina bifida 4D atlas. Details can be found in section Atlas construction.

**Figure 3.**
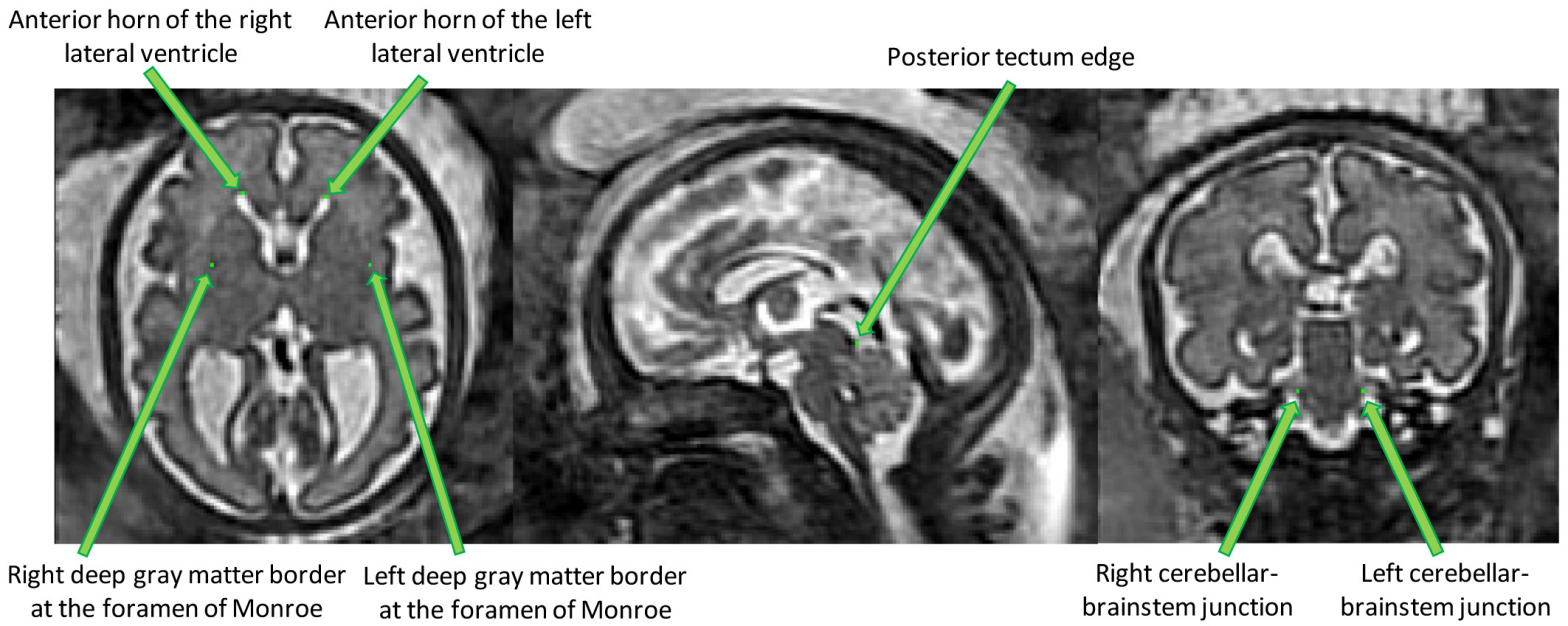
Overview of the proposed anatomical landmarks. Those landmarks were annotated for all the 3D reconstructed magnetic resonance imaging (MRI). They aim at improving the accuracy and the robustness of the image registration steps.

The anatomical landmarks that were selected are: the right and left anterior horn of the lateral ventricles, the posterior tectum plate, the right and left junctions between the cerebellum and the brainstem, and the right and left deep grey matter border at the foramen of Monro. An illustration of those anatomical landmarks can be found in Figure 3.

Those landmarks include anatomical structures that have been reported to be reliably identifiable in the fetal MRI clinical research literature ^28– 30^. Another selection criteria was to choose landmarks that are spread over the fetal brain anatomy to efficiently support image registration. Our proposed annotation protocol can be found in Annotation potocol of anatomical landmarks for fetuses with spina bifida aperta.

The manual annotations of the 90 3D reconstructed MRIs were performed by author EV. Manual annotations of landmarks were performed using the software ITK-SNAP ^31^ version 3.8.0. The annotation of one volume took 12 min on average. It is worth noting, that landmarks can be missing, especially for fetal MRIs before 26 weeks of gestation.

The intra-rater reliability for the anatomical landmarks has been evaluated, as described in Section Intra-rater variability for the annotation of the anatomical landmarks. The proposed anatomical landmarks protocol also included the right and left deep grey matter border at the anterior cavum septi pellucidi line and the right and left deep grey matter border at the posterior cavum septi pellucidi line. However, those landmarks were found to be unreliable and often missing due to the high variation in shape of the cavum septi pellucidi. For this reason, those landmarks were not used for the computation of the atlas but they are present in the annotation protocol. Details can be found in Section Intra-rater variability for the annotation of the anatomical landmarks.

***3.1.5 Age and operation status specific groups of 3D reconstructed MRIs.*** The 3D reconstructed MRIs were grouped with respect to their operation status and their gestational age. Each group of 3D reconstructed MRIs went through the atlas construction pipeline described in section Atlas construction and lead to the computation of a unique volume of our spatio-temporal atlas.

SBA surgery affects the evolution of the fetal brain anatomy ^8, 12, 28^. Therefore, we have chosen to separate the 3D reconstructed MRIs of operated and non-operated fetuses. A group either contains only 3D reconstructed MRIs of fetuses that have been operated for SBA in-utero, or contains only 3D reconstructed MRIs of fetuses that have not been operated.

Each group is assigned with a gestational age ranging from 21 weeks to 34 weeks. Volumes are included in a group only if the gestational age at the time of the acquisition is within 9 days of the gestational age of the group. The description of the cohort used can be found in section Spina bifida aperta cohort used to compute the spatio-temporal atlas and the distribution of gestation ages can be found in Figure 1. As can be seen in Figure 9 and Figure 10, groups for non-operated fetuses cover the gestational ages from 21 weeks to 25 weeks and groups for operated fetuses cover gestational ages from 25 weeks to 34 weeks.

A group is excluded if it contains less than three 3D reconstructed MRIs. In addition, we excluded a group if it did not include both 3D reconstructed MRIs with gestational ages higher and lower than the gestational age of the group. This avoids, for example, to have a group for non-operated fetuses at 26 weeks of gestation that would contain only MRIs at gestational ages 25 weeks or less.

**Data augmentation:** We used right-left flipping as a data augmentation to synthetically increase the amount of volumes in each group. This encourages the atlas to be symmetrical with respect to the central sagittal plane. Right-left flipping has been used in several previous studies on brain MRI atlases ^32, 33^. Imposing symmetry between right and left hemispheres of the atlas volumes aims at reducing potential biases in the cohort used to compute the atlas. In addition, it allows to use the atlas for the study of asymmetry between right and left hemispheres ^33^. Asymmetry between brain hemispheres for normal fetuses has been described as well as the role of hemispheric asymmetry in isolated corpus callosum agenesis. ^34, 35^. To the best of our knowledge, hemispheric asymmetry has not been studied yet in SBA.

### 3.2 Atlas construction

In this section we describe the different steps for the computation of the spina bifida atlas as can be seen in the Initialization and Refinement boxes of the pipeline overview in Figure 2.

***3.2.1 Weighted average of the volumes.*** In this section, we describe the method to average the intensity of 3D reconstructed MRIs after spatial alignment. As described in section Age and operation status specific groups of 3D reconstructed MRIs, data are grouped with respect to their operation status and gestational age. After aligning spatially all the 3D reconstructed MRIs of a group, we average their image intensity to obtain an average fetal brain MRI for the group.

**Weighted average:** To reflect the gestational age associated with each group, we used a time-weighted average. The weight for the volume *i* is defined using a Gaussian kernel as follow


$$\;w_{i}=\frac{1}{\sqrt{2\pi \sigma }}\exp\left(-\frac{1}{2}{\left(\frac{GA_{i}-GA_{target}}{\sigma }\right)}^{2}\right)\;(1)$$

where *GA _target_* is the gestational age of the group and *GA _i_* is the gestational age of volume *i*. The standard deviation value is set to *σ* = 3 days.

In addition, we average each image and its symmetric by right-left flipping to impose to the average volume to be exactly symmetric with respect to the central sagittal plane. This is performed in addition to the data augmentation described in section Age and operation status specific groups of 3D reconstructed MRIs.

Formally, let ${\left\{I_{i}\right\}}_{i=1}^{N}$ be a set of *N* co-registered 3D reconstructed MRIs to average. The weighted average is computed as


$$\;I_{average}=\frac{1}{2N}\sum_{i=1}^{N}w_{i}(I_{i}+S(I_{i}))\;(2)$$

where *S* is the operator that computes the symmetric of a volume with respect to the central sagittal plane.

**Preprocessing:** Before averaging, we transform the intensity of each volume linearly to set the mean (resp. the standard deviation) of the image intensity inside the brain mask to 2000 (resp. 500). Those values were set to approximate the intensity profile of a spatio-temporal fetal brain atlas of normal fetuses ^19^.

***3.2.2 Weighted generalized Procrustes.*** In this section, we describe the optimization method that we used for the joint initial linear alignment of the volumes in a group of 3D reconstructed MRIs. This method is based on a weighted generalized Procrustes method and uses only the anatomical landmarks. Especially, note that the image intensity is not used.

Generalized Procrustes methods ^36^ aims at matching simultaneously *n* configurations of landmarks using linear spatial transformations. Generalized Procrustes methods (without constraints) can be defined as optimization problems of the form ^36, 37^


$$\;\underset{\left\{M_{i},t_{i}\right\}}{\min}\frac{1}{2}{\sum_{i=1}^{n}\sum_{k=1}^{K}\left\|M_{i}x_{i,k}+t_{i}-\frac{1}{n}\sum_{j=1}^{n}\left(M_{j}x_{j,k}+t_{j}\right)\right\|}^{2}\;(3)$$

where *n* is the number of samples, *K* is the number of landmarks, *x*_*i*, *k*_ is the vector of coordinates for the landmark *k* of sample *i*, *t _i_* is the translation for the sample *i*, and *M _i_* is the linear transformation for the sample *i*. In this work we restrict the linear transformations *M _i_* to be anisotropic scaling transformations.

However, for the computation of the spina bifida atlas we have to take into account that landmarks can be missing for some samples. We also would like to weight differently the samples based on their gestational age alike what is done for the weighted average of the 3D reconstructed MRIs in section Weighted average of the volumes.

In this work, we introduce weights in the generalized Procrustes methods. A weight of zeros represents a missing landmark for a sample. The proposed weighted generalized Procrustes method corresponds to the optimization problem


$$\;\underset{\left\{M_{i},t_{i}\right\}}{\min}\frac{1}{2}{\sum_{i=1}^{n}\sum_{k=1}^{K}w_{i,k}\left\|M_{i}x_{i,k}+t_{i}-\frac{\sum_{j=1}^{n}w_{j,k}(M_{j}x_{j,k}+t_{j})}{\sum_{j=1}^{n}w_{j,k}}\right\|}^{2}\;(4)$$

where *w _i_*_,_ *_k_ ≥* 0 is the weight for the landmark *k* of sample *i*. For landmark *k*, sample *i* of gestational age *GA _i_*, and the target gestational age *GA _target_*, we propose to define the weight *w _i_*_,_ *_k_* as


$$\;w_{i,k}=\{\begin{array}{ll} 0 & \text{if}\;\text{landmark}\;\text{k}\;\text{is}\;\text{missing}\;\text{for}\;\text{sample}\;\text{i}\\ 0 & \text{if}|GA_{i}-GA_{target}|\;>3\sigma \\ \frac{1}{\sqrt{2\pi \sigma }}\exp\left(-\frac{1}{2}{\left(\frac{GA_{i}-GA_{target}}{\sigma }\right)}^{2}\right) & \text{otherwise} \end{array}\;(5)$$

The standard deviation value is *σ* = 3 days.

We assume that every landmark was annotated at least once in each group. As a result, $\forall k,\;\sum_{j=1}^{n}w_{j,k}>0$ and the fractions used in ( 4) are well defined.

In general, the optimization problem ( 3) admits an infinity of solutions, including the trivial solution that send all the landmarks to the origin. To tackle this issue, constraints on the size of the system are added ^36, 37^. The optimization problem ( 4) suffers from the same under-specification problem. We therefore choose to constrain the center of mass of the barycenter of the system and the size of the system because it is the most intuitive approach. This leads to the optimization problem


$$\begin{array}{c} \underset{\left\{M_{i},t_{i}\},\{g_{k}\right\}}{\min}\;\frac{1}{2}\sum_{i=1}^{n}\sum_{k=1}^{K}w_{i,k}{\left\|M_{i}x_{i,k}+t_{i}-g_{k}\right\|}^{2}\\ \;\text{s.t.}\;\frac{1}{K}\sum_{k=1}^{K}g_{k}=\frac{1}{K}\sum_{k=1}\frac{\sum_{j=1}^{n}w_{j,k}x_{j,k}}{\sum_{j=1}^{n}w_{j,k}}\\ \;\text{and}\;\frac{1}{K}\sum_{k=1}^{K}{\left\|g_{k}-\frac{1}{K}\sum_{l=1}^{K}g_{l}\right\|}^{2}=\frac{1}{K}\sum_{k=1}{\left\|\frac{\sum_{j=1}^{n}w_{j,k}x_{j,k}}{\sum_{j=1}^{n}w_{j,k}}-\frac{1}{K}\sum_{l=1}\frac{\sum_{j=1}^{n}w_{j,l}x_{j,l}}{\sum_{j=1}^{n}w_{j,l}}\right\|}^{2} \end{array}\;(6)$$

This optimization problem can be solved efficiently using an alternating least squares approach ^37^.

***3.2.3 Non-linear image registration.*** In this section, we describe the non-linear image registration method that we used for the refinement step of the 4D atlas as can be seen in Figure 2. In the refinement step, intermediate atlas MRI volumes have already been computed for all time points. The goal of this step is to improve the image sharpness of the intermediate atlas MRI volumes by registering all the 3D reconstructed MRIs to the intermediate MRI volumes and computing new weighted average volumes using the method described in section Weighted average of the volumes.

We used NiftyReg ^38^ to perform non-linear image registration using image intensity and the anatomical landmarks.

The non-linear image registration optimization problem is the following


$$\;\underset{\text{Θ}}{\min}\;ℒ(I_{subject},\;I_{atlas},\;\phi (\text{Θ}))+R(\text{Θ})\;(7)$$

where *I _subject_* is the 3D reconstructed MRI to be aligned to the 3D atlas time point *I _atlas_* and *ϕ*(Θ) is a spatial transformation parameterized by cubic B-splines of parameters Θ.

The regularization term *R* is a linear combination of the bending energy ^39^ (BE) and the linear energy ^39^ (LE) regularization functions applied to *ϕ*(Θ)


$$\;R(\text{Θ})=\;\alpha_{BE}BE(\phi (\text{Θ}))+\alpha_{LE}LE(\phi (\text{Θ}))\;(8)$$

with *α _BE_* = 0.1 and *α _LE_* = 0.3. More details about the methodology used to tune image registration parameters can be found below.

The data term *ℒ* is a linear combination of the local normalized cross correlation (LNCC) ^40^ and the squared euclidean distances between the landmarks positions


$$ℒ(I_{subject},\;I_{atlas},\;\phi (\text{Θ}))=\alpha_{LNCC}LNCC(I_{subject},\;I_{atlas}\;\circ \phi (\text{Θ}))+\alpha_{LMKS}\;\sum_{k\in {\text{Ω}}_{LMKS}}{\left\|\phi (\text{Θ})(x_{k}^{subject})-x_{k}^{atlas}\right\|}^{2}\;(9)$$

where *Ω _LMKS_* is the set of landmarks that are present for both *I _subject_* and *I _atlas_*, *α _LMKS_* = 0.001 and *α _LNCC_* = (1 – α _LMKS_)(1 – *α _BE_* – *α _LE_*) as implemented in NiftyReg ^38^. The standard deviation of the Gaussian kernel of the LNCC was set to 6 mm. More details about the methodology used to tune image registration parameters can be found below.

**Implementation details:** Registrations that solve the optimization problem ( 7) were computed using the publicly available code for NiftyReg ^38^. We used the latest version of the code on the master branch (git commit *99d584e*). The transformation *ϕ* in ( 7) is parameterized by cubic B-Splines of order 3 with a grid spacing equal to 3 mm. NiftyReg ^38^ uses a pyramidal approach to solve ( 7). We used 3 levels of pyramid which is the default value in NiftyReg. The brain mask were used to mask the voxels outside the brain.

The transformation *ϕ* in ( 7) was initialized with an affine transformation. The affine transformation was computed using a symmetric block-matching approach ^41^ based on image intensities and the brain masks. The implementation of the affine image registration method is included in NiftyReg.

**Parameters tuning:** The parameters *α _BE_*, *α _LE_*, *α _LMKS_*, and the standard deviation of the Gaussian kernel of the LNCC of Equation (8) and Equation (9) were tuned using a grid search. The other parameters of the image registration were not tuned. The values of *α _BE_* were {0.001, 0.01, 0.03, 0.1, 0.3}, the values of *α _LE_* were {0.01, 0.03, 0.1}, the values of *α _LMKS_* were {0.0003, 0.001, 0.003}, and the values for the standard deviation of the LNCC were {1, 2, 4, 6, 8}. We also tried to use the normalized mutual information (NMI) in place of the LNCC. There are no additional hyper-parameters related to NMI.

We selected the best set of parameter values using a subset of 22 pairs of 3D reconstructed MRIs covering the range of gestational ages available. The selection criteria was the mean of the Dice scores for the white matter, the ventricular system, and the cerebellum between volumes after non-linear registration. Details about the segmentation protocol can be found in section Semi-automatic segmentation of the atlas.

It is worth noting that the gradients of the different terms of the objective function in ( 7) have different scales. Therefore, comparing the contribution of the different terms based on their weights is misleading. Our parameter tuning protocol suggests that all the terms of the objective function are important to obtain optimal image registration results.

### 3.3 Semi-automatic segmentation of the atlas

In this section, we describe the semi-automatic method that was used to obtain the segmentation for the proposed spatio-temporal atlas for SBA.

The fetal brains were divided into a total of eight tissue types: white matter (excluding the corpus callosum), ventricular system with the cavum septi pellucidi and cavum vergae, cerebellum, extra-axial cerebrospinal fluid, cortical grey matter, deep grey matter, brainstem, and corpus callosum. A visualization of the segmentations of those tissue types can be found in Figure 9 and Figure 10. The annotation protocol follows the annotation guidelines of the FeTA dataset ^22^. In addition, the corpus callosum was also delineated.

Automatic 3D tissue types probability maps were obtained using a deep learning pipeline trained using partially supervised learning ^24^. An ensemble of ten deep neural networks trained using the Leaf-Dice loss ^24^ has been used. The code and the pre-trained networks used for the automatic segmentation are available here. An average 3D tissue types probability maps for the atlas was obtained using a weighted average method analogous to the one described in section Weighted average of the volumes for the 3D reconstructed MRIs. Formally, let ${\left\{P_{i}\right\}}_{i=1}^{N}$ be a set of *N* co-registered 3D tissue types probability maps to average. The weighted average is computed as


$$\;P_{average}=\frac{1}{2N}\sum_{i=1}^{N}w_{i}(P_{i}+S(P_{i}))\;(10)$$

where *S* is the operator that computes the symmetric of a volume with respect to the central sagittal and the weights *w _i_* are defined as in section Weighted average of the volumes. An initial segmentation of the atlas was obtained using the tissue types of maximum probability for each voxel.

The initial segmentations of the spatio-temporal atlas were quality controlled and corrected when necessary by authors LF and MA, a paediatric radiologist specialized in fetal brain anatomy with eight years of experience in segmenting fetal brain MRIs. Manual segmentations were performed using the software ITK-SNAP ^31^ version 3.8.0.

## 4 Annotation potocol of anatomical landmarks for fetuses with spina bifida aperta

In this section, protocols designed for the selection of imaging landmarks in MRI images of fetal brains with spina bifida aperta (SBA) are outlined. This is aimed to improve the accuracy of image registration. A total of 11 anatomical landmarks per study have been selected for initial assessment. Four in each cerebral hemisphere and three in the posterior fossa.

The first seven landmarks described below were found to be sufficiently reliable. The last four landmarks involving the cavum septi pellucidi were found to be insufficiently reliable.

### 4.1 Anterior horn of the right lateral ventricle

**Figure 4.**
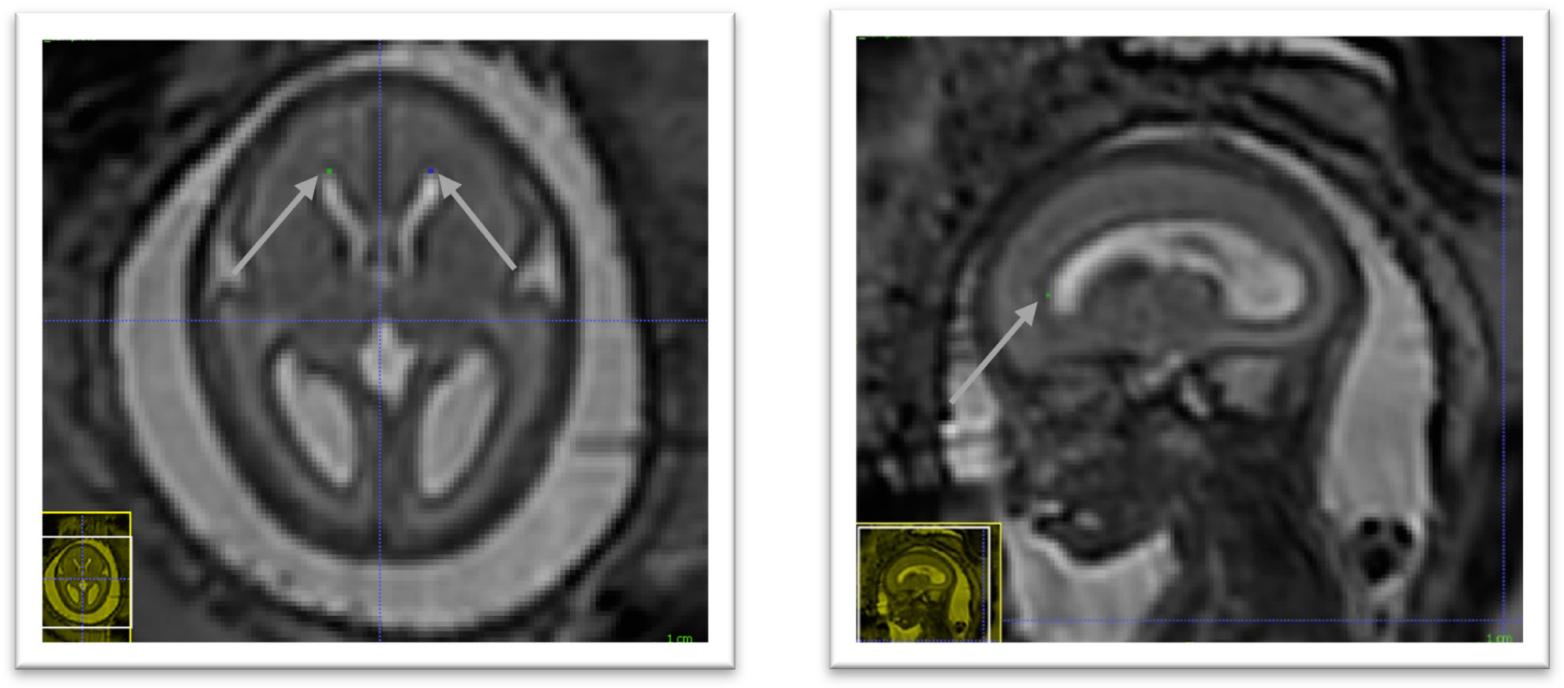
Anterior horn of the right lateral ventricle (green) and anterior horn of the left lateral ventricle (blue).

In the axial plane identify the right lateral ventricle. Use the view in the sagittal plane to select the most anterior slice reached by the ventricle. When this slice is not unique, which occurs when the anterior border of the ventricle is flattened, select the slice at the centre. The border is considered as the brighter intensity value of the two lines of intensity values showing the greatest difference. An illustration is given in Figure 4

### 4.2 Anterior horn of the left lateral ventricle

In the axial plane identify the Left Lateral Ventricle. Use the view in the sagittal plane to select the most anterior slice reached by the ventricle. When this slice is not unique, which occurs when the anterior border of the ventricle is flattened, select the slice at the centre. The border is considered as the brighter intensity value of the two lines of intensity values showing the greatest difference. An illustration is given in Figure 4

### 4.3 Posterior tectum plate

**Figure 5.**
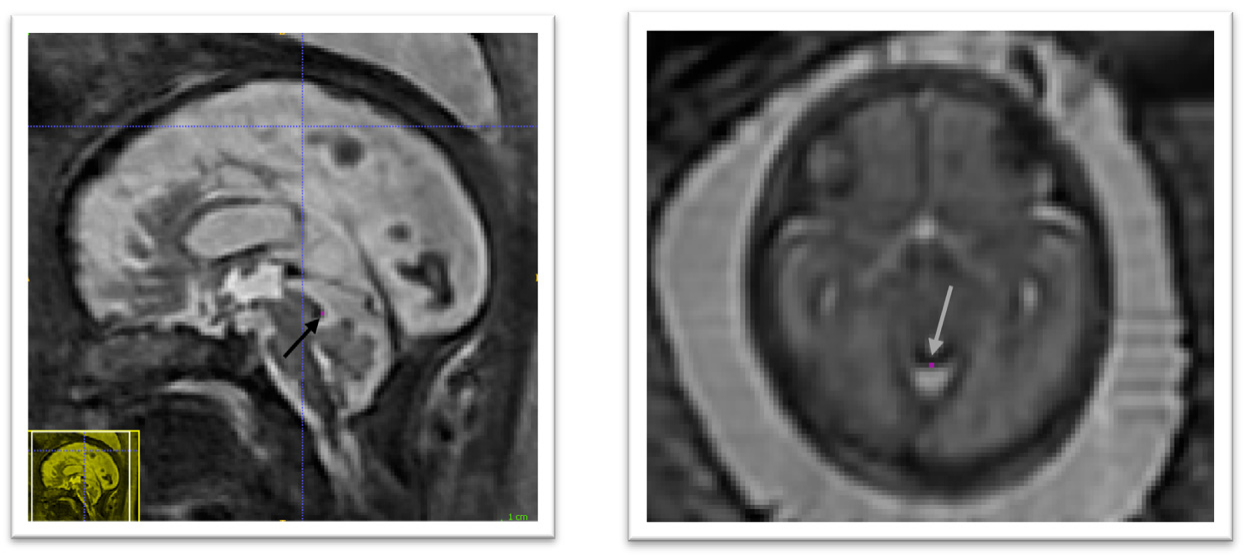
Posterior tectum plate (pink).

Using the sagittal and axial planes locate the tectum. In the axial plane select the midline sagittal slice. Confirm using the sagittal plane that the axial slice is viewing the most prominent part of the tectum. Using the smallest marker select the most posterior point of the tectum tissue. This considered to be the lower intensity value of the two intensity values at the posterior peak showing the greatest difference. An illustration is given in Figure 5

### 4.4 Left cerebellar-brainstem junction

**Figure 6.**
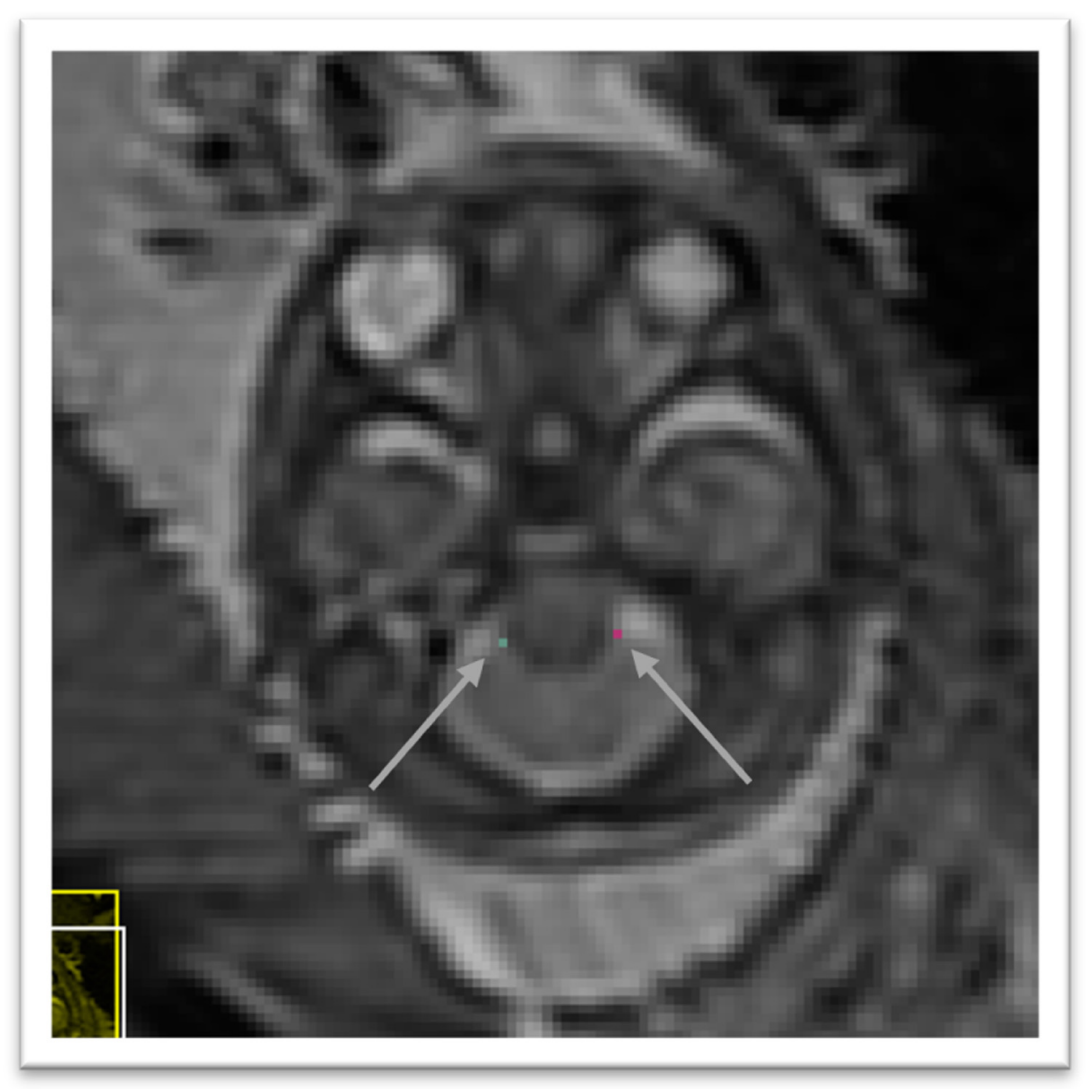
Right cerebellar-brainstem junction (turquoise) and left cerebellar-brainstem junction (pink).

In the axial view we locate the cerebellum and select the slice with the greatest cerebellar width, preferably where the posterior fossa also is seen at its greatest width. The brainstem is found just anterior to the cerebellum and directly meets with the cerebellum along its posterior borders. In this area, we select with the smallest possible marker the most anterior point where the cerebellum and brainstem meet on the left side. The marker should be within cerebellar tissue as oppose to the tissue of the brainstem. An illustration is given in Figure 6

### 4.5 Right cerebellar-brainstem junction

In the axial view we locate the cerebellum and select the slice with the greatest cerebellar width, preferably where the posterior fossa also is seen at its greatest width. The brainstem is found just anterior to the cerebellum and directly meets with the cerebellum along its posterior borders. In this area, we select with the smallest possible marker the most anterior point where the cerebellum and brainstem meet on the right side. The marker should be within cerebellar tissue as oppose to the tissue of the brainstem. An illustration is given in Figure 6

### 4.6 Left deep grey border at foramen of Monro

**Figure 7.**
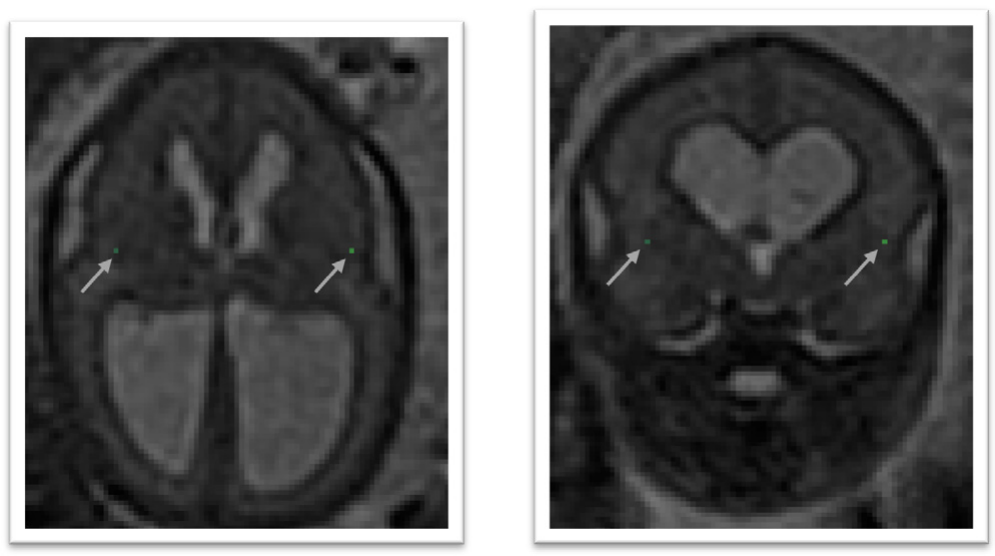
Left deep grey matter border at foramen of Monro (dark olive) and right deep grey matter border at foramen of Monro (lime green).

In the axial view locate the foramen of Monro or the interventricular foramen. The paired foramina connect the lateral ventricles to the third ventricle. The point where the foramina lead into the third ventricle, a horseshoe or trough shaped border is formed anteriorly. If not visible in this way, it can also be observed in the coronal view connecting the anterior horns of the lateral ventricle to the third ventricle. Select the mid-sagittal slice and trace a horizontal line left across from this border. The correct position of the line is considered as the row of brighter intensity value of the two rows of intensity values showing the greatest contrast. The edge of the deep grey matter on the left side which should be visible forming a darker grey arch from the left anterior horn to the left posterior horn of the lateral ventricles. Using the smallest possible marker, mark the edge of the deep grey matter where it intersects with the line. An illustration is given in Figure 7

### 4.7 Right deep grey border at foramen of Monro

In the axial view locate the foramen of Monro or the interventricular foramen. The paired foramina connect the lateral ventricles to the third ventricle. The point where the foramina lead into the third ventricle, a horseshoe or trough shaped border is formed anteriorly. If not visible in this way, it can also be observed in the coronal view connecting the anterior horns of the lateral ventricle to the third ventricle. Select the mid-sagittal slice and trace a horizontal line right across from this border. The correct position of the line is considered as the row of brighter intensity value of the two rows of intensity values showing the greatest contrast. The edge of the deep grey matter on the right side which should be visible forming a darker grey arch from the right anterior horn to the right posterior horn of the lateral ventricles. Using the smallest possible marker, mark the edge of the deep grey matter where it intersects with the line. An illustration is given in Figure 7

### 4.8 Left deep grey border at anterior cavum septi pellucidi line

**Figure 8.**
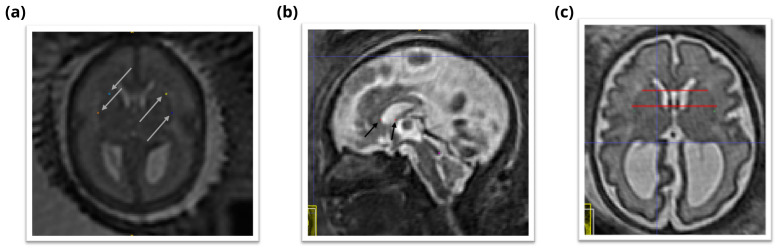
Deep grey matter border with respect to the cavum septi pellucidi (CSP).

In the axial view locate the cavum septi pellucidi (CSP), a cavity in the fetal brain, the leaflets of the septum pellucidum are located between the anterior horns of the lateral ventricles. Select the slice in which the anterior wall of the cavity is found most anteriorly. If there is significant abnormality in this structure it may be helpful to use the sagittal plane to assist in defining this area. Trace a horizontal line left across from the anterior wall of the cavum septi pellucidi. The correct position of the line is considered as the row of brighter intensity value of the two rows of intensity values showing the greatest contrast. The edge of the deep grey matter on the left side forms a darker arch from the left anterior horn to the left posterior horn of the lateral ventricles. Using the smallest possible marker mark the edge of the deep grey matter where it intersects with that line. An illustration is given in Figure 8

**Figure 9.**
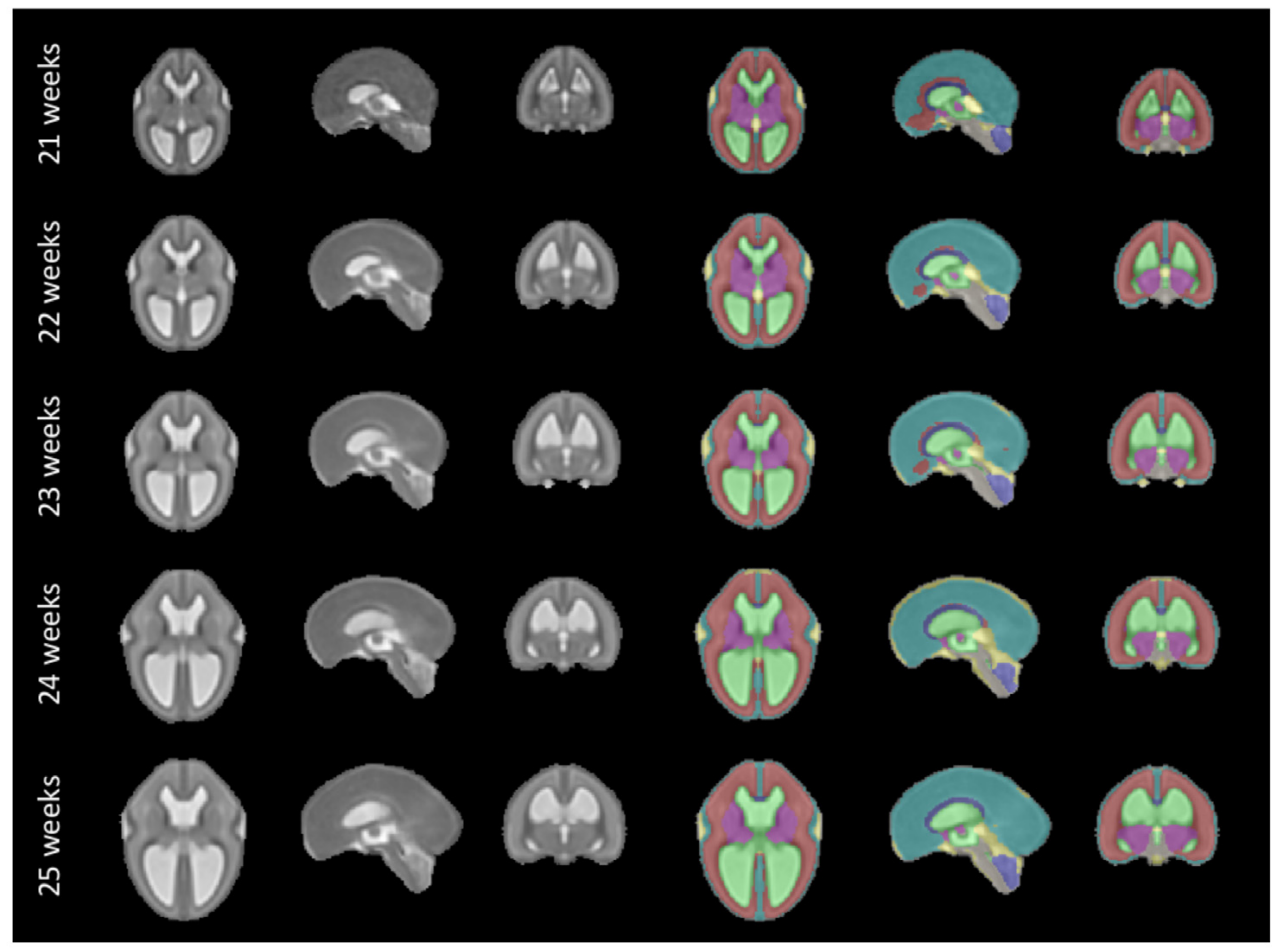
Our spatio-temporal atlas for spina bifida aperta - Part I (not operated). Publicly available here.

**Figure 10.**
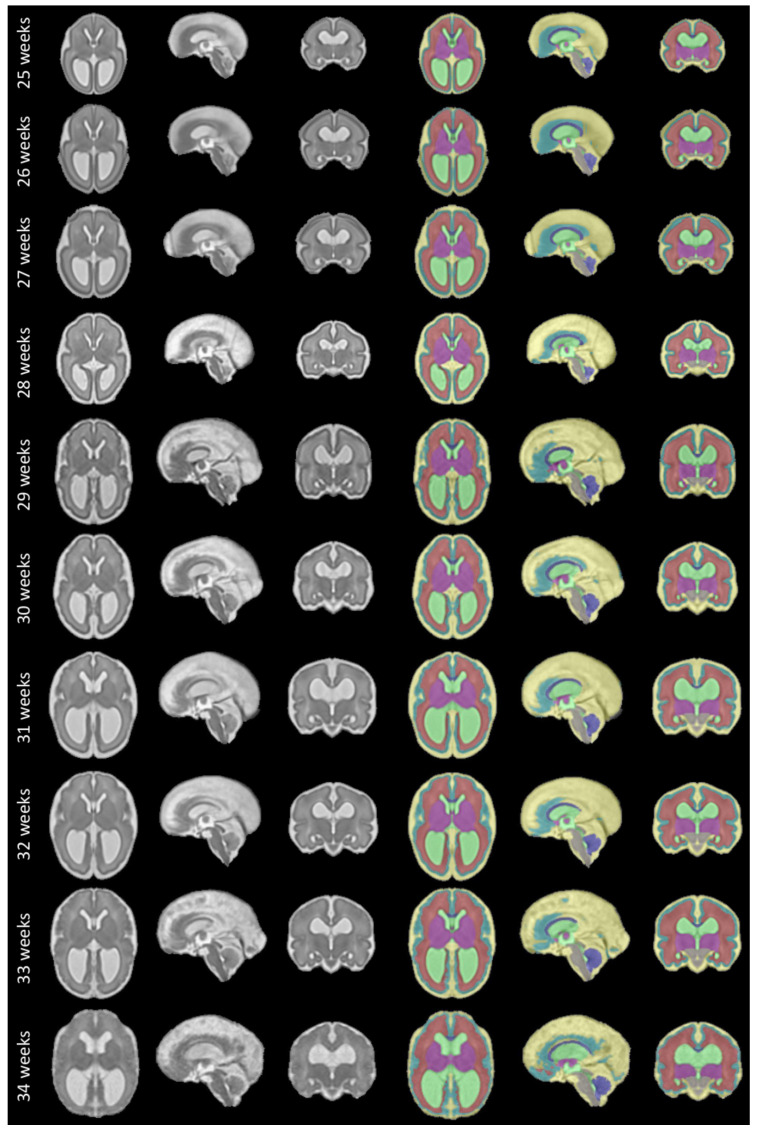
Spatio-temporal atlas for spina bifida aperta - Part II (operated). Publicly available here.

### 4.9 Right deep grey border at the anterior cavum septi pellucidi line

In the axial view locate the cavum septi pellucidi (CSP), a cavity in the fetal brain, the leaflets of the septum pellucidum are located between the anterior horns of the lateral ventricles. Select the slice in which the anterior wall of the cavity is found most anteriorly. If there is significant abnormality in this structure it may be helpful to use the sagittal plane to assist in defining this area. Trace a horizontal line right across from the anterior wall of the cavum septi pellucidi. The correct position of the line is considered as the row of brighter intensity value of the two rows of intensity values showing the greatest contrast. The edge of the deep grey matter on the right side forms a darker arch from the right anterior horn to the right posterior horn of the lateral ventricles. Using the smallest possible marker mark the edge of the deep grey matter where it intersects with that line. An illustration is given in Figure 8

### 4.10 Left deep grey border at posterior cavum septi pellucidi line

In the axial view locate the cavum septi pellucidi, a cavity in the fetal brain, the leaflets of the septum pellucidum are located between the anterior horns of the lateral ventricles. Select the slice in which the anterior wall of the cavity is found most anteriorly. If there is significant abnormality in this structure it may be helpful to use the sagittal plane to assist in defining this area. At this level trace a horizontal line left across from the posterior wall of the cavum septi pellucidi. The correct position of the line is considered as the row of brighter intensity value of the two rows of intensity values showing the greatest contrast. The edge of the deep grey matter on the left side forms a darker arch from the left anterior horn to the left posterior horn of the lateral ventricles. Using the smallest possible marker mark the edge of the deep grey matter where it intersects with that line. An illustration is given in Figure 8

### 4.11 Right deep grey border at the posterior cavum septi pellucidi line

In the axial view locate the cavum septi pellucidi, a cavity in the fetal brain, the leaflets of the septum pellucidum are located between the anterior horns of the lateral ventricles. Select the slice in which the anterior wall of the cavity is found most anteriorly. In this slice trace a horizontal line right across from the posterior wall of the cavum septi pellucidi. The correct position of the line is considered as the row of brighter intensity value of the two rows of intensity values showing the greatest contrast. The edge of the deep grey matter on the right side forms a darker arch from the right anterior horn to the right posterior horn of the lateral ventricles. Using the smallest possible marker mark the edge of the deep grey matter where it intersects with that line. An illustration is given in Figure 8

## 5 Results

### 5.1 Intra-rater variability for the annotation of the anatomical landmarks

To assess intra-rater variability, a subset of 31 3D reconstructed MRIs, selected at random, were marked two times by the same rater, EV. The mean gestational age was 26.2 weeks and the range of gestational ages in the reliability set was 22–34 weeks. Those statistics closely match the one of the full cohort as described in section Spina bifida aperta cohort used to compute the spatio-temporal atlas (the mean gestational age is 26.1 weeks and the range is 21–35 weeks for the full dataset). The two ratings were performed with an interval of at least three weeks to mitigate the bias caused by observer recollection. A landmark was marked absent when the anatomical position described by the protocol was not found within the volume.

The two landmark placements are said to be in agreement if the second landmark placement is inside a 3 × 3 × 3 voxel cube where the original placement is the central voxel. When 95% of the second landmarks fall within this radius, the landmark is considered ‘Excellent’ in terms of intra-rater reliability, when 80% of the second landmarks are in agreement, intra-rater reliability is considered ‘Good’, when 75% fall within the radius of agreement intra-rater reliability is considered ‘Satisfactory’. For landmarks with a probability of agreement of less than 75%, the reliability is considered ‘Poor’. The probabilities that pairs of landmarks are in agreement is estimated based on the assumption that the distribution of distances between first and second marks is Gaussian.

### 5.2 Automatic segmentation of fetal brain 3D MRIs

In this section, we compare the automatic segmentations obtained either using an atlas of normal fetal brains ^19^ or using the proposed atlas for spina bifida aperta (SBA). The quantitative evaluation can be found in Table 2.

We studied the automatic segmentation of fetal brain 3D MRIs into seven tissue types. Fetal brain 3D MRIs from the FeTA dataset ^22^ were used for the evaluation. More details about the dataset used for the evaluation can be found in section Fetal brain 3D MRI used for the evaluation of automatic segmentation.

The automatic segmentations are obtained in two steps: first a volume of the atlas, chosen based on the gestational age, is registered to each fetal brain 3D MRI, and second, after registration, the segmentation of the atlas is propagated. Non-linear image registration is implemented as described in section Non-linear image registration. In particular, we used the same hyper-parameter values. The automatic segmentations for the corpus callosum and the white matter were merged into white matter, since the corpus callosum is part of the white matter segmentation in the FeTA dataset. Automatic segmentations for the SBA cases are computed using either a normal fetal brain atlas ^19^ or our SBA fetal brain atlas as can be seen in the last four rows of Table 2. In addition, we have also compute automatic segmentations for the normal brain cases using the normal fetal brain atlas ^19^ as can be seen in the first two rows of Table 2. The evaluation was performed for each tissue type using the Dice score ^42, 43^ and the Hausdorff distance at percentile 95 ^44^.

## 6 Discussion

**Figure 11.**
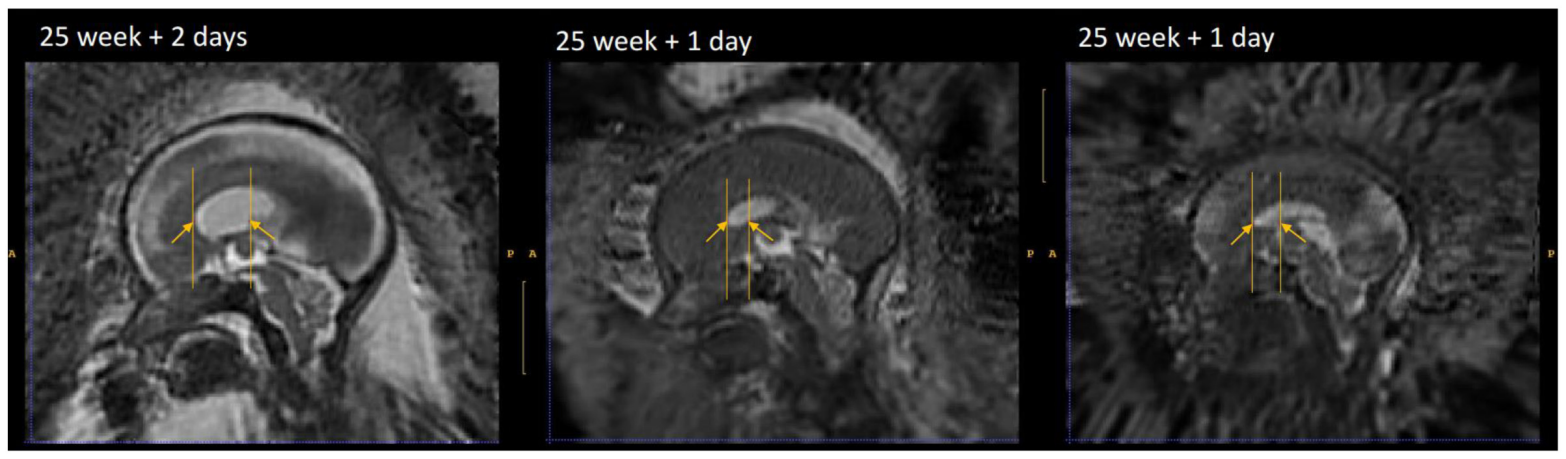
Cavum septi pellucidi (CSP) variation fetuses with 25 weeks of gestation. Yellow arrows indicate the anterior and posterior borders of the CSP as defined by the landmark localisation protocol. This visualisation illustrates the disparity between volumes in terms of shape and size of the CSP.

The proposed spatio-temporal atlas for spina bifida aperta (SBA) is illustrated in Figure 9 and Figure 10 (see *Data availability* ^45^ and *Software availability* for the full atlas). As described in section Spina bifida aperta cohort used to compute the spatio-temporal atlas, the cohort used to compute this atlas contains longitudinal data. This longitudinal dataset of 90 MRIs might be less representative of the whole SBA population than a dataset of 90 MRIs that would contain only singletons. However, the use of longitudinal data adds some implicit temporal consistency in the atlas. The landmarks in the ventricles, the posterior tectum plate, and at the junction of the cerebellar and the brainstem were all found to be reliable enough in terms of distance between successive marks by the same rater as can be seen in Table 1. In addition, those anatomical landmarks were always present, except for the posterior tectum plate that was missing for one reconstructed 3D MRI. However, the landmarks in the deep grey were almost all found to be poorly reliable in terms of distance between successive marks by the same rater. One can group the landmarks in the deep grey matter into two groups: the landmarks based on the foramen of Monro, and the landmarks based on the cavum septi pellucidi. The landmarks based on the foramen of Monro were almost always present. This is in contrast with the landmarks based on the cavum septi pellucidi that were missing up to 29% of the time. In Figure 11, we give an illustration of the anatomical variability of the cavum septi pellucidi in fetuses with SBA. This suggests that the position of landmarks based on the cavum septi pellucidi can vary widely from one subject to the other. As a result, we choose to use the two landmarks based on the foramen of Monro for the computation of the atlas, but to exclude the four landmarks based on the cavum sceptum pellucidum.

**Table 1.** Evaluation of the reliability of the landmarks. We report the estimated percentiles of distances in millimeters between first and second marking for each proposed landmarks. *P*_75_: 75th percentile of distances in millimeters. *P*_80_: 80th percentile of distances in millimeters. *P*_95_: 95th percentile of distances in millimeters. Our reliability score is defined in section 5.1. **LALV**: Anterior Horn of the Left Lateral Ventricle, **RALV**: Anterior Horn of the Right Lateral Ventricle, **PTP**: Posterior Tectum Plate, **LCB**: Left Cerebellar Brainstem Junction, **RCB**: Right Cerebellar Brainstem Junction, **LFOM**: Left Deep Grey Border at Foramen of Monro, **RFOM**: Right Deep Grey Border at Foramen of Monro, **LACSP**: Left Deep Grey Border at Anterior Cavum Septi Pellucidi line, **RACSP**: Right Deep Grey Border at Anterior Cavum Septi Pellucidi line, **LPCSP**: Left Deep Grey Border at Posterior Cavum Septi Pellucidi line, **RPCSP**: Right Deep Grey Border at the Posterior Cavum Septi Pellucidi line.

Landmark	Ratio of Missing (%)	*P*_75_ (mm)	*P*_80_ (mm)	*P*_95_ (mm)	Reliability
LALV	0	1.73	1.95	3.02	Good
RALV	0	1.70	1.91	2.96	Good
PTP	3	1.15	1.29	2.00	Excellent
LCB	0	1.70	1.90	2.95	Good
RCB	0	1.78	2.00	3.10	Good
LFOM	3	2.83	3.17	4.91	Poor
RFOM	0	2.50	2.81	4.35	Satisfactory
LACSP	16	2.74	3.07	4.77	Poor
RACSP	29	2.59	2.91	4.51	Satisfactory
LPCSP	16	3.35	3.76	5.83	Poor
RPCSP	16	3.12	3.50	5.43	Poor

The evaluation of automatic segmentation of fetal brain 3D MRIs in Table 2 suggests that using the proposed atlas for SBA leads to more accurate segmentation of SBA cases than a normal fetal brain atlas. The proposed atlas for SBA outperforms the normal fetal brain atlas in terms of mean Dice scores and mean Hausdorff distances for all tissue types.

**Table 2.** Evaluation of automatic fetal brain segmentation. We report mean (standard deviation) for the Dice score (DSC) in percentages and the Hausdorff distance at 95% (HD95) in millimeters for the seven tissue types. **WM**: white matter, **Vent**: ventricular system, **Cer**: cerebellum, **CSF**: cerebrospinal fluid, **ECSF**: extra-axial CSF, **CGM**: cortical grey matter, **DGM**: deep grey matter, **BS**: brainstem.

Atlas	Cohort	Metric	WM	Vent	Cer	ECSF	CGM	DGM	BS
Normal ^19^	Normal	DSC	87.1 (2.3)	83.8 (4.4)	86.9 (3.7)	86.7 (2.3)	67.9 (5.3)	82.7 (3.2)	81.7 (3.2)
		HD95	1.9 (0.6)	1.2 (0.2)	1.6 (0.4)	1.2 (0.4)	1.5 (0.6)	2.7 (0.7)	2.5 (0.5)
Normal ^19^	Spina Bifida	DSC	69.4 (16.4)	79.9 (8.0)	50.2 (31.3)	49.6 (35.7)	41.7 (21.6)	69.6 (14.5)	62.2 (21.2)
		HD95	4.3 (2.2)	3.5 (2.8)	5.8 (4.2)	10.3 (9.8)	4.1 (2.6)	4.3 (2.5)	3.9 (2.7)
Spina Bifida	Spina Bifida	DSC	80.6 (6.4)	84.0 (10.1)	69.8 (15.6)	54.9 (26.3)	48.8 (15.9)	77.2 (5.1)	71.2 (10.9)
		HD95	3.4 (1.6)	2.0 (1.4)	2.7 (0.8)	9.6 (9.1)	3.2 (1.7)	3.0 (0.7)	3.1 (1.2)

The proposed atlas also leads to lower standard deviations of Dice scores and Hausdorff distances for all tissue types except for the ventricular system. This suggests that automatic segmentation using image registration of an atlas is more robust for SBA when a SBA atlas is used.

In addition, when comparing automatic segmentations of normal fetuses and fetuses with SBA obtained using a normal fetal brain atlas we found a decrease of segmentation accuracy in terms of Dice scores and Hausdorff distances for all tissue types. For the cerebellum, the mean Dice score decreased from 86.9% for normal fetuses to 50.2% for fetuses with SBA. This can be attributed to the Chiari malformation type II which is found in most SBA cases ^2^. The decrease of mean Dice score and the increase of mean Hausdorff distance for the extra-axial cerebrospinal fluid (CSF) can be attributed to the quasi absence of extra-axial CSF in fetuses with SBA.

## 7 Limitations

In this work, we have used MRIs of operated and non-operated fetuses, ie that have or have not undergone fetal surgery to close the spina bifida aperta (SBA) defect in utero. *In-utero* fetal surgery is currently recommended to be performed prior to 26 weeks of gestation. The surgery has been found to influence the evolution of the fetal brain anatomy starting within one week after the operation ^28^. Therefore, a normative atlas for SBA should be computed using only MRIs of non-operated fetuses. This limitation of our work is however due to the clinical data used. To make this limitation clear we have separated the atlas into two parts as illustrated in Figure 9 and Figure 10. This separations is also reflected in the data structure chosen to share the atlas, as detailed in Underlying data ^45^.

In Figure 1, it is worth noting that relatively little cases are available in the range of gestational ages 27 – 31 weeks. As a result, the proposed atlas might be less representative of the SBA population in this range of gestational ages. In particular, this might explain why the ventricle size does not appear to increase linearly for those gestational ages as can be seen in Figure 10.

## 8 Conclusions

In this work we propose the first spatio-temporal fetal brain MRI atlas for spina bifida aperta (SBA).

We propose a semi-automatic pipeline for the computation of spatio-temporal fetal brain atlas. Our pipeline relies on four main components:

• MONAIfbs ^27^, an automatic method for fetal brain extraction in 2D fetal MRIs.
• NiftyMIC ^26^, a 3D super resolution and reconstruction algorithm that allows to obtain isotropic and motion-free volumetric MRIs of the fetal brain.
• A proposed protocol for the annotation of 7 anatomical landmarks in 3D reconstructed fetal brain MRIs.
• A proposed weighted generalize Procrustes method for an unbiased initialization of the atlas based on the anatomical landmarks.

We find that the proposed atlas outperforms a state-of-the-art fetal brain atlas for the automatic segmentation of brain 3D MRIs of fetuses with SBA. This suggests that the proposed atlas for SBA provides a better anatomical prior about the peri-surgical SBA brain. We hypothesise that this atlas could also help improving fetal brain MRI segmentation methods that lacks such prior, such as segmentation methods based on deep learning ^24^. We are planning to investigate this in the future.

## Data availability

### Underlying data

Ethical approval allows us to use the magnetic resonance imaging (MRI) data from University Hospitals Leuven for research and to make publicly available results obtained using those data such as the fetal brain atlas for SBA proposed in this work. The Caldicott guardian at University College London Hospital (UCLH) gave their approval to share the data with University College London and King’s College London researchers for analysis. However, we do not have the required ethical approval to share the original MRI data publicly. Readers and reviewers can email the corresponding author (lucas.fidon@kcl.ac.uk) to request access to the data. Access to the data at UCLH will require approval by the Caldicott guardian at UCLH and access to the data from University Hospitals Leuven will require approval by the ethics committee at University Hospitals Leuven.

The FeTA dataset is publicly available on Synapse: https://doi.org/10.7303/syn23747212. Access requires registration to Synapse and agreement to the terms of use.

The manual segmentations for the fetal brain MRI of FeTA dataset, that we have contributed in our previous work ^24, 25^, are publicly available on Zenodo: https://doi.org/10.5281/zenodo.5148612 under the term of the Creative Commons Attribution-NonCommercial-NoDerivs 3.0 Unported license (CC BY-NC-ND 3.0). Access to the data is restricted. Readers and reviewers can apply for access to the data by filling in a form. The only requirement is to acknowledge that the applicant will not use those data for commercial purposes.

The spatio-temporal atlas of the normal developing fetal brain that we have used for comparison is publicly available at http://crl.med.harvard.edu/research/fetal_brain_atlas/. Access requires readers to fill in an access form. Alternatively, one can download the fetal brain atlas directly from the NiftyMIC GitHub repository.

Zenodo: A Spatio-temporal Atlas of the Developing Fetal Brain with Spina Bifida Aperta. https://doi.org/10. 5281/zenodo.5524312 ^45^.

This project contains the following underlying data:

The project contains 15 folders, each corresponding to a unique volume of our spatio-temporal fetal brain atlas, as illustrated in Figure 9 and Figure 10, and contains four nifti files:

• srr.nii.gz (average 3D reconstructed MRI).
• mask.nii.gz (3D brain mask).
• parcellation.nii.gz (3D segmentation of the fetal brain into 8 tissue types as described in section Semi-automatic segmentation of the atlas).
• lmks.nii.gz (annotations for the 7 anatomical landmarks described is section Anatomical landmarks).

Data are available under the terms of the Creative Commons Zero "No rights reserved" data waiver (CC0 1.0 Public domain dedication). Codes and scripts are available under the terms of the BSD-3-Clause license.

Alternatively, it is possible to download A Spatio-temporal Atlas of the Developing Fetal Brain with Spina Bifida Aperta on Synapse: https://doi.org/10.7303/syn25887675. It is necessary to create a synapse account to be able to download the data.

## Software availability

Source code available from: https://github.com/LucasFidon/spina-bifida-MRI-atlas

Archived source code at the time of publication: https://doi.org/10.5281/zenodo.5524312 ^45^

License: BSD-3-Clause
