# Supplementary material for: A spatio-temporal atlas of the developing fetal brain with spina bifida aperta
Source: Open Res Eur. 2022 Aug 31;1:123. Originally published 2021 Oct 15. [Version 2] doi: 10.12688/openreseurope.13914.2 (PMC10445840; doi:10.12688/openreseurope.13914.2)
Supplement: Peer review history

# Peer review: Reviewer response for version 2

**DOI:** 10.21956/openreseurope.16291.r29995

**Published:** 2022-09-12

**Authors:** Guillaume Auzias

**Affiliations:** 1 Institute for Neurosciences of La Timone, UMR 7289, Aix-Marseille University, Marseille, France

The authors carefully addressed all my concerns.

Is the rationale for developing the new method (or application) clearly explained?

Yes

Is the description of the method technically sound?

Partly

Are the conclusions about the method and its performance adequately supported by the findings presented in the article?

Yes

If any results are presented, are all the source data underlying the results available to ensure full reproducibility?

Yes

Are sufficient details provided to allow replication of the method development and its use by others?

Partly

Reviewer Expertise:

Anatomical MRI data processing and analysis, Fetal MRI

I confirm that I have read this submission and believe that I have an appropriate level of expertise to confirm that it is of an acceptable scientific standard.

# Peer review: Reviewer response for version 1

**DOI:** 10.21956/openreseurope.14998.r28870

**Published:** 2022-04-06

**Authors:** Guillaume Auzias

**Affiliations:** 1 Institute for Neurosciences of La Timone, UMR 7289, Aix-Marseille University, Marseille, France

This work introduces a spatio-temporal atlas specifically designed for fetuses suffering from spina bifida aperta, enabling improved automatic segmentation compared to using an atlas of normal fetuses.

 The description of the entire study is quite complete, with extensive information provided such as the protocol for anatomical landmarks annotation. The proposed atlas is available and easily accessible following the link provided in the article. The efforts for sharing the data and tools are much appreciated.

 While the method is well described and solid, I have few important concerns regarding limitations related to the data available. Also, the motivations for some aspects of the approach are not sufficiently detailed. I believe that addressing my concerns and comments below would clearly benefit the final version of this paper, and would thus contribute to the dissemination of the atlas and tools released.

**Major concern 1**

 The number of subjects involved for the definition of each age-specific atlas can be as small as 3, which is likely to be too small to account for the inter-individual variability. Given the high variability of the brain anatomy in SBA as acknowledged by the authors, 37 subjects might be insufficient to cover the whole spectrum of inter-individual variability and/or the whole spectrum of variations induced by the pathology. More specifically, the atlases at 33 and 34 weeks show a poor delineation and segmentation of the cortical gray matter, which is likely due to the lack of data available to compute the average image. An underestimation of the true inter-individual variance is also possible even when the atlas is sharp. While this is less of a problem for the delineation of the cortical gray matter that is very smooth at early developmental stage, it could affect the delineation of the other structures of great interest such as the ventricles and the cerebellum. This also likely affects the automatic segmentation and might explain the modest performances in particular for the cortical gray matter with a Dice inferior to 50. This is an important limitation for future applications of this atlas that should be explicitly acknowledged and stated in section 7.

**Major concern 2**

 In the proposed approach, the age-specific atlases are built by grouping the fetuses based on their gestational age. This approach suffers from limitations such as the dependency to the number of available cases per age as pointed in my previous concern. Why didn’t you consider using time-varying kernel as proposed in (Serag *et al.*, ref 16 of the submission) to overcome the variations in the distribution of subjects at different ages? I acknowledge this would add some complexity to the weighted generalized Procrustes method described in section 3.2.2, but a sliding window approach as proposed in Serag *et al.* could work, no? More generally, please refer explicitly to Serag *et al.* in Section 3.2 and clarify how your approach relates to that one, that is probably the most closely related publication.

**Major concern 3**

 The sex of fetuses is not considered in the present study. The recent publication (Studholme *et al.*, 2020, see complete ref below) reported different brain growth trajectories between male and female fetuses. The potential implications for the process of atlas building are not straightforward. A study of the influence of sex distribution on such an atlas would be relevant, but I acknowledge it might fall out of the scope of current submission. If the information of fetus sex was recorded, please report the distributions, and if it was not recorded, please mention this absence of information as a limitation the section 7.

 Colin Studholme, Christopher D. Kroenke, and Manjiri Dighe, “Motion Corrected MRI Differentiates Male and Female Human Brain Growth Trajectories from Mid-Gestation,” Nature Communications 11, no. 1 (June 16, 2020): 3038, https://doi.org/10.1038/s41467-020-16763-y.

**Major concern 4**

 The motivations for using anatomical landmarks to constraint the registration are not clear.

 Is the fully automated intensity-driven registration failing? Did you try to adapt the registration algorithm parameters such as e.g. the multi-resolution pyramid? Please add a subsection to state how and why the classical intensity-driven approaches fail in this context.

**Major concern 5**

 Regarding the experiment on the automatic segmentation based on the SBA-specific atlas, an important information is lacking: are the landmarks used for registration when the SBA-specific atlas is used? If the answer is yes, then the comparison with the atlas of normal fetuses is unfair, and an additional experiment without the use of the landmarks with the SBA-specific atlas would be welcome.

**Minor concern 1**

 Make more explicit that steps 3.1.1, 3.1.2 and 3.1.3 of the proposed pipeline are achieved by NiftyMIC on Fig2, e.g. by using a specific color.

**Minor concern 2**

 Please add a small subsection in 3.1.5 providing supplementary information regarding the impact of the surgery on the anatomy of fetuses and comment on how these changes are expected to influence the resulting atlas.

**Minor concern 3**

 In section 3.2.1, the sigma of the Gaussian kernel is set to a value of 3 without any justification.

 Please precise how this parameter value was set and motivate the (expected) limited influence of that parameter on the resulting atlas.

**Minor concern 4**

 An artefact is visible on the 27 week atlas in the orbito-frontal region.

 This artefact does only affect the atlas at that particular age.

 Please investigate and comment.

**Minor concern 5**

 The reference to Table 1 is missing in the text of section 5.1.

**Other minor concerns / Typos**

**In the Plain language summary:**

 “Visualization” is appropriate? -> Understanding?

 “Expected” -> normal?

**In the introduction:**

 “SBA fetuses have also smaller hippocampus7, abnormal cortical thickness and gyrification8,9 , and smaller deep grey matter volume and total brain volume10,11”. References 7, 9, 10, 11 actually involve pediatric or adult populations, while this sentence suggest that all these results were obtained in fetuses, which is thus incorrect. Please be more specific in separating the studies on fetuses or on post-natal data.

 “Atlases can also be used to measure variability in the brain anatomy of an individual as compared to the whole population” to the whole population -> to the model supposed to be representative of the whole population.

 Those anatomical landmarks are used for two things in our pipeline -> please rephrase.

Is the rationale for developing the new method (or application) clearly explained?

Yes

Is the description of the method technically sound?

Partly

Are the conclusions about the method and its performance adequately supported by the findings presented in the article?

Yes

If any results are presented, are all the source data underlying the results available to ensure full reproducibility?

Yes

Are sufficient details provided to allow replication of the method development and its use by others?

Partly

Reviewer Expertise:

Anatomical MRI data processing and analysis, Fetal MRI

I confirm that I have read this submission and believe that I have an appropriate level of expertise to confirm that it is of an acceptable scientific standard, however I have significant reservations, as outlined above.

## References

[1] : Motion corrected MRI differentiates male and female human brain growth trajectories from mid-gestation. *Nature Communications* .2020;11(1) : 10.1038/s41467-020-16763-y 10.1038/s41467-020-16763-y PMC729799132546755

# Response

**Published:** 2022-08-24

**Authors:** Lucas Fidon

**Affiliations:** 21503, UK

We thank the reviewer Guillaume Auzias for his insights and comments. They have allowed us to improve the manuscript and our atlas. We respond below to the concerns of the reviewer one by one.

**Major concern 1** While limited, the current number of subjects included in the atlas construction already allowed us to reach useable performance as illustrated in our evaluation section. Increasing this number would obviously be of interest but was found to be impractical within the scope of this project. From a methodological perspective, we have mitigated the low number of subjects for some gestational ages using Gaussian kernel regression (please see equation (1) in the revision) and right-left symmetry (please see 3.1.5 Data augmentation in the revision). We give more details about this in our response to major concern 2 below.  To better analyze the segmentation performance per gestational age, we have added the fetal brain 3D MRIs from the FeTA dataset release 2 to the segmentation evaluation cohort. This brings the total of MRIs of neurotypical (resp. spinal bifida) cases used in our evaluation to 32 3D MRIs (resp. 36 3D MRIs).  Results in Table 2 in the revision have been updated and confirm that the proposed fetal brain atlas for spina bifida outperforms a state-of-the-art neurotypical fetal brain atlas in terms of mean Dice scores for all tissue types for spinal bifida cases.  In addition, we have added two new figures (see Fig. 9 and Fig. 10 in the revision) with boxplots of the Dice scores and Hausdorff distance for each method, each tissue type, and each gestational age (in weeks) separately.  

 Those figures are only for the spina bifida cases of the evaluation cohort (n=36).  Regarding the cortical gray matter of spina bifida cases specifically, the boxplots show that the Dice scores are lower and the Hausdorff higher at earlier developmental gestational ages. This suggests that contrary to the hypothesis of the reviewer, the smoothness of the cortical gray matter is not the anatomical feature dominating the difficulty of the automatic segmentation of the cortical gray matter.  Besides, the proposed fetal brain atlas for spina bifida outperforms the neurotypical atlas for the cortical gray matter for two segmentation metrics and all gestational age, except for 27 weeks.  It is worth noting that the gestational ages range for evaluation is 20 weeks to 30 weeks for spina bifida. This is because there are no MRIs of spina bifida cases older than 30 weeks at the time of acquisition in the FeTA dataset. In particular, the atlases at 33 and 34 weeks have no influence on the Dice scores reported in the paper. We have clarified this in the dataset description. This is also a limitation of our evaluation and we have added it in the limitations section.  We agree with the reviewer that the cortical segmentation at 33 and 34 weeks was rather poor, especially at the insula in both weeks and in the occipital and inferotemporal cortical lining at 34 weeks. Co-author Michael Aertsen has now corrected these segmentations and we have updated the atlas online.  We do acknowledge that our open-source segmentations for the proposed spina bifida atlas might remain suboptimal for some applications. We would like to emphasize that we have made the atlas and the segmentation available under the license CC0 1.0 Public domain dedication to allow other researchers to improve and redistribute the segmentations if they wish.

**Major concern 2** We apologize for the confusion and clarify that, similar to Serag et al., we already use time-varying kernels in our approach. The time-varying kernel is defined in equation (1) of paragraph 3.2.1 in the revision. The time-varying weights defined in (1) are used at two stages of the computation of the atlas: 

 1. for the initialization of the age-specific atlases in the generalized Procrustes method (see equations (4), (5), and (6)) 

 2. in every update of the age-specific atlases (see equation (2)).  We think paragraph 3.1.5 in the initial manuscript about the age-specific groups might have been confusing. We have clarified the paragraph:  “Each group is assigned with a gestational age ranging from 21 weeks to 34 weeks. Volumes are included in a group only if the gestational age at the time of the acquisition is within 9 days of the gestational age of the group.”   By adding:  “This implies that there are overlaps between groups. For example, the 24 weeks group contains the fetal brain MRIs acquired between 22 weeks + 4 days and 25 weeks + 3 days of gestation. In addition, the contribution of each volume within an age-specific group is weighted using a time-varying Gaussian kernel, as defined in the next section in (1). The value of 9 days, used above, is chosen to correspond to 3 x sigma where sigma is defined in the time-varying Gaussian kernel regression (1).”  To improve clarity, in section titles and in fig. 2, we have also replaced “weighted” by “time-weighted”.  We have also added a citation to Serag et al before equation (1) in the revision.

**Major concern 3** A figure showing the distribution per gestational age of the genders of the fetuses whose 3D MRIs were used to compute the atlas has been added in Fig. 1 (right part) of the revision.   We have added in the limitations:  “The proposed atlas mixes male and female fetuses. However, recent work reported different brain growth trajectories between male and female neurotypical fetuses (Studholme et al., 2020). Sex-specific atlases may be of interest to better represent the populations of male and female fetuses with spina bifida aperta.”  We thank the reviewer for the suggestion.  Despite only females being present between 28 weeks and 30 weeks of gestation, globally we have found no statistical difference between the distributions of gestational ages for males and females using a Mann-Whitney U test with a confidence level of 95% (p-value=0.35).

**Major concern 4** We have several motivations that support the use of the proposed anatomical landmarks for computing the atlases.  Firstly, without an informative initialization, we found that intensity-driven registration cannot cope with the large variability observed in fetal imaging for spinal bifida. Then, the Procrustes method used for the initialization of the atlases could not be performed without the anatomical landmarks since it uses only the landmarks and not the intensity (please see equations (3) to (6) in the revision).  Secondly, in the paragraph parameter tuning of section 3.2.3 in the revision, we give details of the grid search of the hyper-parameters that we have conducted for the non-linear registration. We conclude this paragraph with “Our parameter tuning protocol suggests that all the terms of the objective function are important to obtain optimal image registration results.” This includes the term related to the anatomical landmarks. We have clarified this by adding:  “In particular, this supports the usefulness of the landmarks for the registration since a non-minimal value of alpha_LMKS was optimal.”

**Major concern 5** We considered that our landmarks would not typically be available at test time for the purposes of atlas-based segmentation. The landmarks are thus not used during registration for the evaluation of automatic segmentation. We have clarified this in section 5.2 of the revision.

**Minor concern 1** We have updated Fig 2 in the revision as requested.

**Minor concern 2** We have added a paragraph about this in the discussion:  “With fetal surgery, the open neural tube defect is closed and thus the continuous leakage of cerebrospinal fluid (CSF) is stopped. This leads to an increase in CSF within the skull, surrounding the cerebrum and cerebellum, leading to a better distinction of the grey matter from the inner lining of the skull. This may explain the higher segmentation accuracy for the grey matter after 27 weeks as from this timepoint the evaluation cohort includes only postoperative images.   In addition to the increase in CSF within the skull, the closure of the defect leads to a reversal of the hindbrain herniation, as demonstrated in a previous paper by our group this happens already within 7 days after surgery in the majority of cases (Aertsen et al, 2019 AJNR). The reversal of the hindbrain information in combination with the increase in fluid surrounding the cerebellum and brainstem in the posterior fossa, improves the distinction of the cerebellum and brainstem from the skull base. Again we notice an improved performance after 27 weeks confirming the impact of fluid restoration in the skull on our automatic segmentation algorithm.”

**Minor concern 3** We have chosen the value sigma = 3 days so that an interval [-sigma, sigma] covers approximately one week which is the time unit for the atlases. We have not tested other values of sigma and we have not evaluated the influence of sigma on the resulting atlas. This is now clarified in the revision, please see 3.2.1.

**Minor concern 4** We think that this artefact is due to variability in the topology of the extra-axial cerebrospinal fluid (CSF) at week 26 weeks for operated fetuses. At this time point, the extra-axial CSF can be either circumferential or limited as illustrated in Fig. 14 that we have added in the revision. In this case, the diffeomorphic hypothesis of our non-linear registration step is violated and can lead to such artefacts. It is worth noting that this issue affects the atlas at 27 weeks due to the use of a time-weighted regression kernel.  We have investigated the segmentation performance per tissue type and per gestational age in Fig 9 and Fig 10 of the revision. Week 27 is the only week for which the proposed atlas underperforms the baseline for the extra-axial CSF and the deep grey matter. The artefact in the orbito-frontal region may account for the suboptimal automatic segmentation at 27 weeks.  In addition, it is worth noting the large variability of the segmentation results for week 26 in Fig. 9 and Fig.10 of the revision for the two atlases. This can be attributed to the variability in the topology of the extra-axial CSF illustrated in Fig. 14 of the revision. At week 26, the spina bifida atlas performs best on 3D MRIs of fetuses with limited extra-axial CSF (Fig. 14 of the revision left) while the neurotypical atlas performs best for 3D MRIs of fetuses with circumferential extra-axial CSF (Fig. 14 of the revision right).  We have added the comments above to the discussion and the limitations.

**Minor concern 5** We have added the missing reference in section 5.1 of the revision.

**Other minor concerns / Typos** Typos were corrected, thank you. We have clarified the use of some references in the introduction:  “In postnatal life, children and adults with spina bifida aperta are known to have smaller hippocampus7, abnormal cortical thickness and gyrification8,9 , and smaller deep grey matter volume and total brain volume10,11. In a small pilot study, it has been observed that fetal brain volume and shape is different after spina bifida repair compared to controls (Mufti et al, neuroradiology, 2021)”

# Peer review: Reviewer response for version 1

**DOI:** 10.21956/openreseurope.14998.r27829

**Published:** 2021-11-26

**Authors:** Andras Jakab

**Affiliations:** 1 Center for MR-Research, University Children's Hospital Zürich, Zürich, Switzerland

This article describes the first publicly available spatio-temporal fetal cerebral MRI atlas for spina bifida. The atlas comprises anatomical templates and multi-tissue segmentation maps for different gestational ages and operated vs. non-operated fetuses. The manuscript adequately describes the methods that were used to create the dataset. One novelty is that the work used anatomical landmarks and a weighted generalized Procrustes method for the temporal regularization of data. I believe that the atlas will be a valuable resource for the quantitative, automated and large-scale analysis of SBA MRI data.

 The data is high quality and the methods are reproducible. As the authors point out, the work has a few weaknesses. For example, the atlas is based on low case numbers for the more advanced pregnancies. To cover this age range, the authors may consider including postnatal images since many of these infants are born prematurely. SBA fetuses have a large variability in terms of their ventricular width. In the clinical practice, cases with much larger ventricles as the mean template in the Atlas are common. The analysis of these cases might be problematic. Perhaps in the future, the authors could also consider generating anatomical templates with a varying level of hydrocephalus.

 I would suggest that the authors consider citing the final FeTA article instead of the pre-print: https://doi.org/10.1038/s41597-021-00946-3

Is the rationale for developing the new method (or application) clearly explained?

Yes

Is the description of the method technically sound?

Yes

Are the conclusions about the method and its performance adequately supported by the findings presented in the article?

Yes

If any results are presented, are all the source data underlying the results available to ensure full reproducibility?

Yes

Are sufficient details provided to allow replication of the method development and its use by others?

Yes

Reviewer Expertise:

Fetal and infant neuroimaging research. Group leader / PI.

I confirm that I have read this submission and believe that I have an appropriate level of expertise to confirm that it is of an acceptable scientific standard.

## References

[1] : An automatic multi-tissue human fetal brain segmentation benchmark using the Fetal Tissue Annotation Dataset. *Scientific Data* .2021;8(1) : 10.1038/s41597-021-00946-3 10.1038/s41597-021-00946-3 34230489PMC8260784

# Response

**Published:** 2022-08-24

**Authors:** Lucas Fidon

**Affiliations:** 21503, UK

We thank the reviewer András Jakab for assessing our work and for his feedback. We have updated the reference to the FeTA dataset as suggested.
